# Optimizing Post-acute Myocardial Infarction Left Ventricular Thrombus Care: When, How, and How Long

**DOI:** 10.1016/j.jacadv.2025.102423

**Published:** 2025-12-05

**Authors:** Dimitrios Kotzadamis, Efstathios Pagourelias, Theodoros Karamitsos, Vasilios Vassilikos, Georgios Giannopoulos

**Affiliations:** a Hippokration Hospital, Aristotle University, Thessaloniki, Greece; b AHEPA Hospital, Aristotle University, Thessaloniki, Greece

Left ventricular thrombus (LVT) secondary to acute myocardial infarction (AMI) is a recognized complication, traditionally treated with vitamin K antagonists. In spite of widespread direct oral anticoagulants adoption, randomized trials remain scarce. Therefore, we commend Shah et al who demonstrated that rivaroxaban is noninferior to warfarin succeeding even earlier complete LVT resolution.^1^ However specific aspects of this study merit closer scrutiny.

Despite appropriate emphasis on early detection, the inclusion criterion requiring LVT identification within 7 days after AMI is overly restrictive, as it excludes a substantial number of patients who may develop thrombus beyond the first week.^2^ Accordingly, a follow-up imaging assessment at a later interval might have identified additional LVT cases eligible for anticoagulation.

Although acknowledged as a limitation, the imaging strategy also raises some concerns. Echocardiography without contrast agent administration likely reduced LVT detection, as contrast echocardiography may double sensitivity.^3^ Moreover, cardiac magnetic resonance remains the diagnostic gold standard offering superior sensitivity and specificity, even for the detection of small or mural thrombi, often missed by echocardiography, presenting however significant thromboembolic risk.^4^ Thus, greater reliance on advanced imaging modalities should be warranted.

A final consideration is treatment duration, as all patients, irrespective of clinical background, received 4 weeks of triple antithrombotic therapy (TAT), followed by 8 weeks of dual antithrombotic therapy. Given that, 44.4% of participants had diabetes—a high thrombotic risk subgroup—contemporary guidance suggests considering TAT extension up to 3 months when thrombotic risk predominates.^5^ Hence, assessing whether a prolonged TAT regimen performs even better in terms of thrombus resolution among diabetic patients with AMI and LVT would be informative for practice.
